# Nudging Immunity: The Case for Vaccinating Children in School and Day Care by Default

**DOI:** 10.1007/s10730-019-09383-7

**Published:** 2019-10-12

**Authors:** Alberto Giubilini, Lucius Caviola, Hannah Maslen, Thomas Douglas, Anne-Marie Nussberger, Nadira Faber, Samantha Vanderslott, Sarah Loving, Mark Harrison, Julian Savulescu

**Affiliations:** 1 Wellcome Centre for Ethics and Humanities, University of Oxford, Oxford, UK; 2 Oxford Martin School, University of Oxford, Oxford, UK; 3 Department of Experimental Psychology, University of Oxford, Oxford, UK; 4 Uehiro Centre for Practical Ethics, University of Oxford, Littlegate House, 16-17 St Ebbes St, OX1 1PT Oxford, UK; 5 Oxford Vaccine Group, University of Oxford, Oxford, UK; 6 Wellcome Unit for the History of Medicine, University of Oxford, Oxford, UK; 7 Murdoch Children’s Research Institute, Melbourne, Australia

**Keywords:** Vaccination, Nudging, Vaccination policies, School vaccination, Anti-vaxxers

## Abstract

Many parents are hesitant about, or face motivational barriers to, vaccinating their children. In this paper, we propose a type of vaccination policy that could be implemented either in addition to coercive vaccination or as an alternative to it in order to increase paediatric vaccination uptake in a non-coercive way. We propose the use of vaccination nudges that exploit the very same decision biases that often undermine vaccination uptake. In particular, we propose a policy under which children would be vaccinated at school or day-care by default, without requiring parental authorization, but with parents retaining the right to opt their children out of vaccination. We show that such a policy is (1) likely to be effective, at least in cases in which non-vaccination is due to practical obstacles, rather than to strong beliefs about vaccines, (2) ethically acceptable and less controversial than some alternatives because it is not coercive and affects individual autonomy only in a morally unproblematic way, and (3) likely to receive support from the UK public, on the basis of original empirical research we have conducted on the lay public.

## Introduction

Some people do not believe in phenomena many others take as facts, like climate change or the efficacy and safety of vaccines. In a similar vein, some people fail to make choices that are consistent with the best evidence available. For example, some refuse to vaccinate their children and are convinced this refusal is in their children’s best interest, while scientific evidence and expert opinions strongly support the opposite view. Why do people neglect or deny scientific truths and/or fail to make decisions based on them? There are different possible answers to this question that appeal to different psychological factors, some of which we will explore below. For instance, people tend to resort to their pre-existing world views in justifying and making sense of what they ultimately do or do not do. This can be problematic if pre-existing worldviews involve misconceptions (e.g., “vaccinations are dangerous and ineffective”). Another explanation is that people are often subject to cognitive and decision biases, which might lead them to disregard or misinterpret available evidence that would be incorporated differently under careful deliberation.

The relevance of psychological factors in vaccination decisions is supported by a time-series analysis of global vaccination coverage (De Figueiredo et al. 2016), which showed that vaccination uptake in high-income countries such as the United Kingdom, European, and North American countries is not associated with socio-economic factors, while socio-economic factors are strongly, positively correlated with vaccination uptake in less developed regions of the world. This points towards non-structural barriers to vaccination, such as risk perceptions or philosophical beliefs, playing a crucial role in vaccination uptake in developed countries (Larson et al. 2014). In the following, we will discuss how psychological factors such as biased (risk) perceptions and motivated reasoning contribute to vaccination hesitancy and we will argue that these warrant interventions that target the psychology of vaccination decisions. More precisely, in this paper, we will argue that a potentially effective and ethically acceptable vaccination policy is a form of nudge whereby children are vaccinated in school by default without parents’ explicit consent, but where parents can actively opt out if they so wish. As we will suggest, this policy might be effective at raising vaccination uptake while preserving parents’ autonomy regarding vaccinating their children.

When we write of vaccination in this paper, we refer to the vaccines typically recommended or mandated by most developed healthcare systems along with the timings and doses of these, such as the measles, mumps, rubella (MMR) vaccine, the 6-in-1 vaccine, the flu vaccine, and others. There is no doubt that “[v]accines are one of the greatest success stories in public health” (Centers for Disease Control 2015). Much of vaccinations’ success can be attributed to negligible rates of adverse complications (Navin 2015, p. 6). The side-effects of vaccines tend to be mild rather than serious (e.g., reactions at the injection site). More serious side effects are uncommon or rare, and the most severe are so infrequent that they are difficult to assess statistically (World Health Organization 2018a). Take, for instance, Guillain-Barré syndrome—a serious autoimmune disorder that can lead to paralysis—which has shown some association with the flu vaccine. However, not only is the syndrome’s actual causal link with vaccines subject to debate among experts (Centers for Disease Control 2018), but even assuming a causal link with the flu vaccine, the frequency is in the order of one case in every one to 2 million doses. By contrast, even in a developed country like the UK 600 people die on average every year from complications of seasonal flu (Oxford Vaccine Group 2018). As with this example, minor risks related to vaccination are vastly outweighed by the benefits it confers. For instance, it is probably more likely to suffer Guillain-Barré syndrome as a result of *infection* with influenza than as a result of vaccination (Centers for Disease Control 2018). Similarly, skin rashes or febrile seizures—two rare symptoms that have been triggered by the MMR vaccine—are actually significantly more common in cases of measles. Additionally, infectious diseases (even seemingly trivial ones such as the flu) can have serious complications, including fatal ones. Despite the effectiveness and safety of vaccinations, many parents hesitate or refuse to allow their children to be vaccinated against diseases such as measles, mumps, rubella, flu, and other diseases for which paediatric vaccination is typically recommended (or in some cases mandated). Scepticism towards vaccines may, in turn, fuel opposition to coercive vaccination policies. Quite apart from that, vaccine hesitancy and refusal contribute to the death toll of infectious disease. For instance, largely because of resistance to vaccination, 72 people died only in Europe of measles (World Health Organization 2018b).

Empirical work suggests that convincing people to vaccinate their children is often not a matter of educating them by providing more factual information. For example, although clinicians are often advised to discuss the risks and benefits of vaccination with hesitant parents (Omer et al. 2009), arguments based on risk–benefit analysis have been shown to be unlikely to convince hesitant parents to vaccinate their children (Meszaros et al. 1996). Nyhan et al. (2014) found that this was the case in relation to messages designed to reduce misperceptions about the MMR vaccine’s safety and to increase its uptake.

The reasons why people oppose or are skeptical about vaccines are manifold (see, e.g., Hough-Telford et al. 2016; Harmsen et al. 2013; Largent 2012; Smith et al. 2011). Religious beliefs often play a role, as the recent outbreak in Rockland County in New York among ultra-Orthodox communities (153 cases) suggests.

However, even when opposition to vaccines is not motivated by religious beliefs, vaccine refusal tends not to occur simply because of parental ignorance. On the contrary, “vaccine refusers tend to know more about vaccines than parents who vaccinate do, even if vaccine refusers often have some false beliefs too” (Navin 2015, p. 10). Moreover, providing pro-vaccination information in the context of a polarized debate may appear adversarial to parents who have questions and concerns about vaccination (Vanderslott 2019). In fact, strong negations of vaccine-adverse events have been shown to increase perceived risks associated with vaccination (Betsch and Sachse 2013).

If the provision of information concerning vaccination is not sufficient to overcome vaccine hesitancy and refusal, could other interventions increase vaccination coverage? One possible and much discussed solution is the use of coercive measures, such as making certain vaccinations a school-entry requirement. There is an ongoing debate about the ethics, effectiveness, and necessity of compulsory vaccination. For instance, one could argue that vaccination could be coerced on the basis of harm prevention (e.g., Flanigan 2014; Pierik 2018), as a matter of fairness in the distribution of the burdens entailed by the realisation of herd immunity (Giubilini 2019), or as a matter of paternalism (Dare 2009). However, as a matter of fact, many people, including those who are more explicitly in favour of mandatory vaccination (e.g., Flanigan 2014; Pierik 2018), disagree with coercive vaccination policies unless they are strictly necessary to guarantee herd immunity, often by appealing to the principle of least restrictive alternative (PLRA) in public health. According to the principle of the “least restrictive alternative” (Nuffield Council on Bioethics 2007; Childress et al. 2002, p. 173; Gostin 2008, p. 142), less restrictive solutions are ethically preferable as long as they are effective in realising the relevant public health goal. Suppose the relevant public health goal is the realisation of herd immunity, which is defined as a situation in which enough people in a given population are vaccinated against a certain disease so that unprotected individuals are very unlikely to become infected: is there a way to realise herd immunity without resorting to compulsion, even if this means not getting all outright vaccine refusers to vaccinate their children? If so, the PLRA would require adopting such solution. For example, in the case of measles, herd immunity is achieved when 92–95% of the population is immune, and in the case of rubella the required coverage rate is 83–90%. Assuming the proper target of a vaccination policy is herd immunity, a small minority could become a problem only if they are clustered together and so an outbreak among those people is more likely, or if they have a high influence in changing the views of the wider population.

Thus, it might be sufficient and even efficient (Laesk 2011) to ignore a small minority of outright vaccine refusers and to target so called “vaccine-hesitant” parents who put off, spread out, or pick-and-choose vaccinations, or those who do not vaccinate for trivial reasons. As the SAGE Working Group on Vaccine Hesitancy describes it, “vaccine hesitancy refers to delay in acceptance or refusal of vaccination despite availability of vaccination services” (Macdonald and the SAGE Working Group on Vaccine Hesitancy 2015, p. 4163). Because this definition of vaccine hesitancy is very broad in that it presents vaccine refusal as an instance of vaccine hesitancy, we will adopt a narrower definition that allows us to keep it distinct from outright vaccine refusal: in this paper, we understand vaccine hesitancy as a delay in acceptance of vaccination or as a failure to vaccinate amongst parents who have a weak preference for not vaccinating their children, rather than a strong intractable or ideological opposition to vaccines (as is instead the case with vaccine refusal).

Vaccine-hesitant parents are the most suitable target of the non-coercive vaccination policy we are presenting in this paper, namely a vaccination “nudge”. Nudges can be defined as “interventions that steer people in particular directions but that also allow them to go their own way.” (Sunstein 2015, p. 417). Cass Sunstein gives examples of reminders, warnings, and default rules as nudges. The important aspect of a nudge, in the context of policy making, is that it “must not impose significant material incentives (including disincentives)” (Sunstein 2015, p. 417) and that it must preserve freedom of choice. As such, the nudging intervention we propose would often be the least restrictive effective means to ensure herd immunity and, indeed, could provide a highly efficient strategy for achieving herd immunity without exerting any form of coercion.

In the next section, we describe some of the biases that can affect vaccination decisions. In “Coercion and the Principle of the Least Restrictive Alternative in Public Health”, we introduce some key concepts of public ethics that are relevant for reflection upon vaccination policies. Finally, in “Default Vaccination and its Ethical Justification”, we bring these lines of discussions—psychological and ethical—together and suggest that a potentially effective and ethically acceptable vaccination policy is a form of opt-out vaccination at school, based on an opt-out or presumed consent system. We will also present an empirical study that we have conducted, which suggests that the public in the UK would be supportive of this type of vaccination policy.

## Vaccination Decisions and Biases

Psychological research suggests that decision biases, and particularly omission bias and default effects, play an important role in vaccination decisions and often cause people not to vaccinate. Omission bias is “the tendency to see a negative outcome resulting from inaction (omission) as more favourable than the same negative outcome resulting from action (commission)” (DiBonaventura and Chapman 2008, p. 2; also see Spranca et al. 1991). The tendencies in question are not necessarily irrational and they might survive careful reflection. However, we follow the psychological literature in referring to these tendencies or preferences as “biases”.

DiBonaventura and Chapman (2008) have demonstrated that omission bias—as well as other biases like the naturalness bias—predicts flu vaccination intentions and actual vaccination behaviour in adults. In their study, they presented participants with a hypothetical scenario stipulating a 10% chance of contracting an infectious disease. Participants then indicated whether they would accept a vaccination that had some risk of causing the same disease as a side-effect. A substantial proportion of participants rejected a vaccination coming with a chance lower than or equal to 9% and thus showed omission bias in these scenarios. Importantly, the degree to which participants showed omission bias in the scenarios translated into real-world behaviour, namely a lower uptake of a free vaccination conveniently offered at the workplace.

Similar findings have been obtained by other studies that tested omission bias in the case of childhood vaccination (Ritov and Baron 1992; Asch et al. 1994). For example, Asch and colleagues found that omission bias does play a role in decisions not to vaccinate one’s children against pertussis. Interestingly, and consistent with the considerations made above about scientific literacy and vaccination decisions, they also found that “[t]he association between nonvaccination and omission bias is not peculiar to those with more or less education, although the more educated respondents (…) were more likely to resist vaccination” (Asch et al. 1994, p. 121). The explanations of some participants in the studies by Ritov and Baron (1992) highlight how parents perceived harm that could occur as a result of their decision to immunise their child (commission/action) as less acceptable than harm that could occur as a result of not immunising their child (omission/inaction). For instance, here is what a subject reported: “‘I feel that if I vaccinated my kid and he died I would be more responsible for his death than if I hadn’t vaccinated him and he died—sounds strange, I know. So I would not be willing to take as high a risk with the vaccine as I would with the flu.’ Another subject wrote, ‘I’d rather take my chance that the child will not catch the flu than to be responsible for giving my child a vaccine which could be fatal.’ A third wrote, ‘… I did not want to risk killing the child with a vaccine that is optional. It would have been my fault if the child died from the vaccine’” (Ritov and Baron 1992, p. 275).

A related psychological factor that has been shown to influence vaccination decisions is the so called “default effect”, defined as “the tendency for decision makers to stick with the default, or the option that takes effect if one does not make an explicit choice” (Li and Chapman 2013, p. 190). This is sometimes called ‘status quo bias’. A powerful demonstration of the default effect’s influence on health-related behaviour in general is that organ donation rates are substantially higher in countries with opt-out policies, where people are presumed to consent to donating their organs after death unless they declare otherwise (Johnson and Goldstein 2003). A study by Chapman et al. (2010) found that the default effect also influences individual vaccination decisions: two groups of university employees received an email informing them that free flu shots were available. For one (opt-in) group, the email also contained a link to make an appointment to receive the shot, while the other (opt-out) group was informed that their appointment had already been scheduled and were given the option to cancel the appointment. The result was that 45% of those in the opt-out group received the flu shot, compared to 33% of those in the opt-in group (Chapman et al. 2010). It is interesting to note that default effects are partly driven by perceived social norms; i.e., people infer that the default option is the norm, that therefore others will take it too, and that others think one should take it (Everett et al. 2015).

All these empirical observations suggest that vaccine hesitancy, if not vaccine refusal, can at least be partly explained by omission bias and the default effect or by a combination of these. In light of the reviewed evidence, it is plausible to suppose that if being vaccinated against certain infectious diseases did not require individual action but rather was the status quo, more children, including in particular those of vaccine-hesitant parents, would get vaccinated.

## Coercion and the Principle of the Least Restrictive Alternative in Public Health

One way of addressing the problem of vaccine hesitancy and of vaccine refusal as we have already alluded to is to use some form of coercion. There are various philosophical and common-sense understandings of coercion (e.g., Nozick 1969; Held 1972; Feinberg 1989; Frankfurt 1988; Wertheimer 1987/89; O’Neill 1991; Beauchamp and Childress 2001, p. 95), but a comprehensive overview of these views is beyond the scope of this paper. Here, we will simply assume a conception of coercion understood as an action through which an agent leaves another agent with “no reasonable choice” or “no acceptable alternative” (Wertheimer 1989, pp. 30, 36–37) but to do X, when the second agent would otherwise prefer not to do X and when the first agent acts with the intention of inducing the second agent to do X. On some more restrictive accounts, only threats of penalties can be coercive (e.g., Nozick 1969; Wertheimer 1987/89), while according to others offers of irresistible incentives can also be coercive (Held 1972; Feinberg 1989; Frankfurt 1988). Here, we are concerned only with coercion brought about through threats of penalties; for example, preventing families from enrolling non-vaccinated children in day-care or schools or fining parents of non-vaccinated children.

Are coercive policies morally acceptable, when they are effective in achieving a desirable public health goal such as herd immunity? That depends on what the alternatives are. As mentioned above, a principle commonly invoked in public health ethics is the principle of the least restrictive alternative (Childress et al. 2002, p. 173), or PLRA. According to the PLRA, “if two interventions can both efficaciously and effectively address a public health or health policy issue and are equal in all other morally relevant respects, the intervention least restrictive of personal liberties ought to be preferred” (Saghai 2014, p. 350). According to Lawrence Gostin, the PLRA requires a “government to utilize the policy that achieves the public health objective, with the least intrusion on personal rights and freedoms” (Gostin 2008, p. 142), i.e., to implement “the least intrusive and burdensome policy” (Gostin 2008, p. 68). Thus, non-coercive policies are ethically preferable to coercive ones, *other things being equal*.

We can assume that the level of restrictiveness of a policy is a function of at least three parameters: the degree or risk of harm imposed, limitation of autonomy, and limitation of freedom. It is plausible to claim that the risks of vaccines are sufficiently low to justify vaccinating children in order to bring about herd immunity (Giubilini 2019). What we are more interested in here are the limitations of autonomy and of freedom that a nudge-based vaccination policy might entail, compared to a coercive policy. A minimal definition of autonomy, which we will accept, is the one according to which autonomy consists in self-determination (Ploug and Holm 2015, p. 28) in a broad sense of the term: I am autonomous if I can act based on my personal beliefs and desires (there is a further philosophical and psychological question as to what counts as my authentic belief and desire, but we can leave this issue aside for the purpose of the present paper). Freedom can be taken to indicate the capacity to act according to one’s autonomous decision without external constraints, such as the use of force or threats by another agent. Thus, by compromising freedom through external constraints such as threats of significant penalties, a coercive policy also compromises autonomy because it prevents a person from acting based on their personal preferences or desires and replaces these with the desire to simply avoid a penalty.

Freedom is one, but not the only, determinant of autonomy. For instance, a policy can infringe upon individuals’ autonomy without compromising individual freedom, because a person might be tricked into performing a certain action (thus not exercising her autonomy) even if the alternatives remain easily accessible. Nudges work in a related way. A nudge is a way of structuring the range of options that are available to a person, or the “choice architecture”, in such a way as to sway a person’s choices by utilizing certain biases and heuristics that characterize human decision making, without the need to provide people with reasons or to enforce coercive measures. Importantly, nudges do not (1) attach significant costs or burdens to any of the alternatives, (2) constrain the range of options that are open to a person, or (3) conceal relevant factual information (Thaler and Sunstein 2008, p. 5; Blumenthal-Barby and Burroughs 2012, p. 3). Nudges are less restrictive than mandates or penalties because, unlike mandates and penalties, they are not coercive and do not limit freedom: nothing in the concept of nudging suggests that certain options are rendered unreasonable to refuse. Conditions (1) and (2) are what differentiate nudges from coercive threats. For instance, to anticipate our proposal, suppose we implement a policy whereby children are vaccinated at school by default without parents explicit consent, but with the possibility for parents to opt out by signing an exemption form. In such a case, there is no strong incentive that would make it unreasonably difficult or costly for parents to opt their children out of any vaccination that is scheduled at school.

Admittedly, nudges can to a certain extent diminish individuals’ autonomy. For instance, nudges that exploit default effects can intrude on autonomy because of “the force of inertia” on individuals’ decision making (Sunstein 2015, p. 438). But, these types of nudges leave individuals free to choose any option they want, because there are no external constraints on their choice and do not generally render any of the options unreasonably costly or difficult. So, nudges are not coercive in this sense.

On the other hand, a policy that compromises individual freedom will also often compromise autonomy^1^ by closing off certain options or making them extremely unappealing when an individual would otherwise have preferred to choose those options. Prohibiting school enrolment to children who are not vaccinated (as is the case for example in the US) or depriving non-vaccinating parents of child care benefits (as is the case in Australia) would often prevent individuals who would otherwise have chosen not to vaccinate their children from doing so, and thus arguably limits their ability to autonomously decide how their children are treated. Such policies compromise autonomy by compromising freedom because parents opposed to vaccination would ultimately vaccinate their children not out of their personal desires and beliefs but merely in response to an imposed necessity to avoid the penalty. Because parents who are opposed to vaccination, if they decided to nonetheless vaccinate their children, would do so out of a desire to avoid the penalty instead of out of their personal desires and beliefs, such policies compromise autonomy by compromising freedom. Thus, some policies that compromise individual freedom through threats of penalties are coercive in a way in which policies that only undermine internal bases of autonomy, such as some instances of nudging, are not.

If at least equally effective, nudging would therefore under one respect be ethically preferable to the introduction of mandatory vaccination because, by infringing at most, and only to a limited degree, upon autonomy without compromising freedom, it would be less restrictive than mandatory vaccination and it would be noncoercive, thus complying with the PLRA on the assumption that no even less restrictive effective alternative is available. In particular, it would be less restrictive than currently accepted school exclusion or withholding of child care benefits policies. We also want to suggest—although this is an empirical claim for which empirical evidence will be needed—that there are reasons to think that the form of nudging we are proposing could be effective, and it could further strengthen the effectiveness of mandatory vaccination policies by utilizing the very same biases or tendencies that have been associated with reduced vaccination uptake and that we have described above. It might be thought that the fact that a nudge deploys certain biases or unconscious tendencies is somewhat ethically problematic. However, there are three reasons why nudging vaccination is nevertheless ethically acceptable, all things considered.

First, because the nudge is used to the benefit of individuals and the community (Halpern et al. 2013). It is often justified to restrict a person’s autonomy when this benefits the community at large regardless of any benefit to the restricted person, as in cases of quarantine and isolation (Giubilini et al. 2018), but the case is even stronger when there plausibly is a benefit to the persons whose autonomy is restricted. (In this case, the parents nudged towards vaccinating their children, who will benefit from having a child who is more likely to stay healthy.)

Second, because, as we will see in more detail below, to the extent that the nudge undermines individual autonomy to a certain degree, it does so in an ethically unproblematic way, i.e., in circumstances in which respect for individual autonomy is not as ethically relevant as it normally is.

Third, because it is likely that exploiting the biases in the desired way would lead people to make choices they would not object to when deliberating under careful (as far as possible “bias-free”) reflection, as is likely to be the case with vaccination. Thus, in an important sense of ‘autonomy’, nudging would often promote, rather than hinder, autonomy. After all, it is true that nudging mostly works by exploiting people’s irrationality, at least “in so far as what is driving my action does not constitute a reason for my action—i.e., it is not a feature of the action that I endorse as a feature that makes the action desirable” (Bovens 2009, p. 210). However, nudging can lead people towards options that are in their own best interest and therefore that they would pick if they were to choose under careful deliberation, at least given certain assumptions about human rational decision making. Above, we have defined autonomy as self-determination based on one’s own beliefs and desires, but it is plausible to assume that people desire to make decisions that are in their own best-interest, or at least in their own perceived best interest, and that are as informed as possible. Some people would still want to refuse vaccination even if nudged to vaccinate because they would still think that vaccination is not in their and their children’s best interests. But, many people whose vaccination decisions are determined by omission bias and default effect would not want their choices to be influenced by those effects. In such cases, it seems that complying with the nudge would be promoting, rather than hindering, their autonomy, as we have defined it here.

One of the typical arguments in favour of nudging policies is that some form of default “choice architecture” is inevitably present and that there is no neutral choice architecture (Thaler and Sunstein 2008). In most countries, the present architecture is one where the default is non-vaccination. It would be better to choose the default option that is likely to have the overall better consequences and that is likely to lead people to make the kind of choice they would make if they were to decide under careful reflection.

## Default Vaccination and its Ethical Justification

In their seminal work on nudging, Richard Thaler and Cass Sunstein refer to the principle that ethically justifies nudging as “libertarian paternalism”: the “libertarian” aspect of nudging lies in the idea that people remain free to do what they wish in the sense that all the options remain open and easily accessible to them; the paternalistic aspect “lies in the claim that it is legitimate for choice architects to try to influence people’s behaviour in order to make their lives longer, healthier, and better” (Thaler and Sunstein 2008, p. 5).

An example of a nudge in the context of vaccination policies is the use of framing devices, such as presenting the risks and benefits of vaccination in a way that emphasize the latter rather than the former; for instance, instead of telling parents that 0.001% of vaccinated children experience serious side-effects, we could provide them with the factually equivalent information that 99.999% of vaccinated children do not have serious complications (Navin 2017, p. 45). In this way, we would not be concealing or misrepresenting any factual information, but we would only be framing it in a way that makes it more likely that parents will base their decision on the positive aspects of vaccination. Other forms of nudging that have proven to be effective in convincing people to choose vaccination include, as far as adult vaccination is concerned, playing on “implementation intentions”, e.g., asking individuals to write down the date and time at which they plan to visit on-site vaccination clinics at their employer’s firm (Milkman et al. 2011), and invoking social norms, e.g., telling people that almost everyone else in their community is vaccinated (Li and Chapman 2013; Navin 2017).

Here, we want to propose an alternative form of nudging that holds promise for being effective in diminishing nonmedical exemption requests where vaccination is mandatory and in increasing vaccination uptake where vaccination is not mandatory. The type of nudge we have in mind consists in vaccinating children at school or day-care by default; for example by having doctors or nurses visiting institutions and carrying out the inoculations *without seeking the explicit consent of parents*. Instead, the parents would simply be informed of the vaccination and of its benefits and risks, requested to bring forward any contraindications to vaccination (such as immunoincompetence), which could also be recorded at enrolment), and would be given the possibility to opt out if they so wished. We will refer to this type of policy as “default vaccination”. This type of policy, like all nudges (according to the definition we have provided above), does not (1) significantly change the incentives or disincentives attached to the different options, (2) constrain the range of options that are open to a person, or (3) conceal relevant factual information. When the parents are informed that the vaccination will take place, they have the choice whether to allow it to happen or to opt out. The fact that allowing the vaccination to take place requires them to do nothing can sway their choice towards vaccination without any threat or constraint, that is, in a non-coercive way.

Paradoxically, some of the same biases and heuristics that sway parents’ decisions away from vaccination, with our proposed default vaccination might do the exact opposite; i.e., they might redirect parents’ decisions towards having their children vaccinated.

Let us start with omission bias. We might hypothesize—although an empirical validation would be required to confirm the hypothesis—that omission bias would facilitate the effectiveness of default vaccination. This is because, where vaccination at school or day care is the default option, if parents do nothing, the child is vaccinated. With vaccination being the result of an omission, namely the omission to opt-out, any (unlikely) negative outcomes resulting from this omission would likely be discounted in comparison to negative outcomes resulting from a commission, which in this case would be opt-out of the default policy by issuing an exemption request.

Default vaccination also would exploit the “force of inertia” in people’s psychology of decision making: since people often tend to stick with the default, making vaccination the default option is likely to result in more vaccinations at least among parents who are hesitant about vaccines, but who do not have deeply held beliefs against vaccination.

Now, default vaccination at school or day care, like many other forms of nudging, might well undermine autonomy, as we said above. However, when it does, we suggest that the autonomy infringement is not significantly morally problematic. Our model of default vaccination would only affect the vaccination decisions of parents with relatively weak preferences against vaccination and who would otherwise not vaccinate their children even if they do not oppose vaccination in principle—i.e., some of those we have identified as “hesitant” parents, rather than as outright “vaccine refusers”. This group includes, for instance, parents who would otherwise not vaccinate their children because they do not think it is worth making time for it, do not want to go through the inconvenience of paying a visit to the doctor, simply think that their child is healthy enough and there is no need for vaccination, do not want to make the effort to fill in and return a form to their child’s school, or only have very mild concerns about vaccines. According to the definition of autonomy we have provided above, even choices based on such weak preferences would count as autonomous, even if such preferences are explained by omission bias or default effect. In fact, at least some exemptions from child vaccination mandates are obtained for reasons of mere convenience. This is suggested by the fact that school-based immunization clinics have proven to be effective in reducing exemption rates and therefore in increasing the number of fully immunized students (Wang et al. 2014, p. e80; Sadaf et al. 2013, p. 4297). However, those with very strong views against vaccination would normally have the cognitive and motivational resources to resist the psychological effects of nudging. Their autonomy would be preserved. As explained by Yashar Saghai, there is sufficient psychological evidence to believe that “at least when individuals have strong enough preferences, goals, or beliefs, they are likely to become aware of an anomaly” (Saghai 2013, p. 489), i.e., of a discrepancy between their conscious desires and what they are nudged to do. Such awareness would probably enable them to inhibit the automatic cognitive process that the nudging would otherwise exploit. Thus, if I am strongly opposed to vaccination because I believe that it is unsafe or because I do not think that my child should be subject to a (alleged) risk in order to benefit others, then the fact that vaccination is the default option would not convince me to vaccinate my child. I would almost certainly still request an exemption for my child. So, even assuming that default vaccination would to some extent undermine people’s autonomy, it would normally undermine the autonomy only of those who do not have a strong interest in making an autonomous choice with regard to vaccinating their children. Therefore, arguably, the violation of autonomy entailed by nudging does not seem to be morally problematic.

Some authors have discussed some forms of “default vaccination” in the context of school (Opel and Omer 2015; Reiter et al. 2012). Opel and Omer (2015) have described school-entry vaccination requirements with opt-out procedures in the US as forms of “default” vaccination, but we are proposing that the school, rather than the parents, would take responsibility for ensuring that the vaccination takes place. According to our proposal, parents would be able to opt-out not of the vaccination “requirement” (as in the US), but of vaccination taking place at school. Reiter et al. (2012) have studied the potential effects on parents’ consent of default vaccination policies through hypothetical scenarios involving HPV vaccination in schools for boys and young men. They concluded that “parents’ consent to HPV vaccination through school-located vaccination programs may be higher with opt-in policies and when offering HPV vaccine along with other recommended adolescent vaccines” (Reiter et al. 2012, p. 656). These results are necessary to justify an opt-out school-based vaccination policy like the one we are proposing because they suggest that this policy is likely to be effective at raising vaccination uptake. However, they are not sufficient as it would remain to be shown that such policy is ethically justified. In this paper, we have provided such ethical justification.

Importantly, a survey we conducted (see “Survey on UK Citizens’ Support of Vaccination Policies in Schools”) suggests that the public in the UK would be supportive of this type of policy. While not an indicator of moral justification, public support displays the pragmatic feasibility of such an intervention. Besides, many countries, such as Britain, have a long (post-1948) tradition of providing vaccinations to school-age children, which illustrates the potential of schools as a focus for campaigns to encourage vaccination and perhaps as a locus for immunization of children who missed vaccination as infants. Our proposal represents a way of better exploiting the potential of school-based immunization programs for improving vaccine uptake. Also, thanks to the success of vaccination, by the 1960s parents were less inclined to think of tuberculosis as a major threat to their children’s health and rumours about the harmful side-effects of vaccination attracted greater attention. This probably contributed to several tuberculosis outbreaks during the 1960s in the UK, such as the one that occurred in Stafford in 1961, in which 26 cases occurred among children attending a local grammar school. These lapses were ascribed to low vaccination coverage and might have been prevented if an opt-out system had been in operation, rather than the policy of parental consent for each vaccination administered.

A possible objection to default vaccination has to do with moral responsibility and legal liability for possible serious side-effects of vaccines. Although serious side-effects are extremely rare, they could occur. The higher the number of children that are vaccinated, the more likely the occurrence of serious side-effects. A policy that succeeds in increasing vaccination rates will also increase the risk that someone will experience the serious side-effects of vaccines. But, if a child is vaccinated without parents’ explicit consent and then experiences a serious side-effect, the question of legal liability may arise. While liability of governments and pharmaceutical companies for any injury after consent is often covered through national compensation schemes (Mello 2008), liability when parents have not consented to vaccination is a different matter.

With this in mind, we should consider that if we are concerned about attribution of moral responsibility and legal liability for harm arising from vaccination, we should be equally, if not more (given the higher risks), concerned about attribution of moral responsibility and legal liability in the case of non-vaccination. For instance, 1 in every 10 children with measles will get ear infections that can result in permanent hearing loss, 1 in every 20 gets pneumonia, and 1 or 2 in every 1000 people with measles die (Centers for Disease Control 2017); besides, measles entails a risk of seizures that is higher than the risk of seizure entailed by the MMR vaccine (NHS 2015). But, parents are not normally held legally responsible when such outcomes are the result of a precise choice not to vaccinate their children or in any case of a blameworthy omission. This asymmetry in attribution of responsibilities might simply reflect omission bias, or might instead reflect the fact that people have good reasons to think that the act-omission distinction has some moral relevance. In any case, it seems that in the case of health policies with small individual costs and large individual and collective benefits, the minimization of the risk and of the magnitude of harm for individual children and the population as a whole should be a relevant consideration. Perhaps, we want to suggest, it should be more relevant than the consideration about who or which entity is ultimately responsible or liable for that harm.

And after all, does the state really need to be held morally responsible and legally liable? We think the answer is negative. Let us assume that not opting out does not count as valid consent by parents (after all, not opting out might simply be a consequence of a failure of communication between the school and parents), and therefore that the responsibility for vaccine injuries is not on parents. Why think that the responsibility—moral and legal—should then be transferred to the state? There is no reason to think that responsibility and liability are zero-sum games (Giubilini and Levy 2018); sometimes, unfortunately, bad things happen without sufficient grounds for holding any individual or institution morally or legally responsible. If a state has good reasons to enforce a certain policy—including a strong enough ethical justification—it is not necessarily the case that it should be held morally and legally responsible for the possible negative effects of the policy. Victims of vaccine injuries or their parents in the case of child vaccination are arguably entitled to some form of compensation (Mello 2008), as is already the case in many countries (Looker and Kelly 2011), quite independently of whether vaccination was coerced, nudged, or a completely autonomous choice. But, it is a mistake to think that a right to compensation for the negative side effects of a policy implies some form of moral or legal responsibility on the part of the government that implemented that policy, if the government was all things considered ethically justified in implementing that policy. Actually, the very notion of vaccine injury compensation funds suggests that the responsibility to compensate victims of vaccine injuries falls on society at large rather than on the specific government that implemented a certain vaccination policy.

## Survey on UK Citizens’ Support of Vaccination Policies in Schools

In a survey, we empirically investigated the extent to which UK citizens would support different vaccination policies in school. More specifically, we were interested in people’s views on policies regulating measles, mumps, and rubella (MMR) vaccination in secondary schools in the UK that vary in how strict they are. By strictness, we mean the extent to which vaccination is presented as the default option or even mandatory, and the ease with which parents can choose another option. The level of strictness in vaccination policies reflects a point of tension between respecting a parent’s right to decide whether they want to vaccinate their children, against the public health objective of lowering infection rates and achieving herd immunity. We tested five different policies from least strict (Policy A) to most strict (Policy E):

(A) *No Vaccination* MMR catch-up vaccines are not offered at secondary schools.
(B) *Consent* MMR catch-up vaccines are offered at secondary schools with explicit permission of the parents. Accepting the vaccine for the child is voluntary, and parents must give their written permission. Under this policy, children will not receive the vaccine in school unless their parents explicitly say they want this to happen.
(C) *General Permission* MMR catch-up vaccines are offered at secondary schools with general permission of the parents in advance. Schools have a policy of advising parents that when the child enters school all recommended vaccines, including MMR catch-up for those not sufficiently vaccinated, will be administered at a later date. Parents can give permission at the point of enrolment, but this permission can be retracted at any time if the parent changes their mind. Under this policy, all those children whose parents have explicitly said they want their child to receive vaccinations during secondary school years will receive all the vaccines in school, unless the parent later explicitly withdraws their permission.
(D) *Opt*-*Out* MMR catch-up vaccines are offered at secondary schools unless parents opt out. Accepting the vaccine for the child is voluntary, and parents must indicate if they do not give permission. Under this policy, all children who need the vaccine will receive the vaccine unless their parents explicitly say they do not want this to happen.
(E) *Mandatory* MMR catch-up vaccines are required at secondary schools. It is mandatory for all children to receive the vaccine unless there is a medical reason against it. Parents have no choice but to accept this.

Our hypothesis was that support for policy D (Opt-Out) would be highest because it leaves parents the freedom to opt out, while still creating a choice architecture that encourages high vaccination rates. Previous research on nudging has shown that people are generally sympathetic to Nudges (default options) in many different domains (Sunstein et al. 2017), especially when the desired outcome is in line with most citizens’ values. Importantly, we predicted that the people who were worried about the risks of vaccination would be less likely to support these stricter policies.

## Methods

Five hundred and four British participants took part in the study online via the platform Prolific, and received £0.70 payment for their participation. Forty seven participants were excluded for failing an attention check question, leaving a final sample of 457 participants (344 female; *M*_age_ = 38.34, *SD*_age_ = 12.56). Participants also provided a range of other demographic information: 61.9% of participants had children (youngest child age *M* = 10.01, *SD* = 10.70). 91% of these participants indicated their children were fully vaccinated. 90.4% of the participants themselves said they were fully vaccinated. Participants indicated they were relatively low in religiosity (*M* = 2.23, *SD* = 1.70 on a 7-point rating scale) and slightly more politically liberal than conservative (*M* = 3.49, *SD* = 1.34 again on a 7-point rating scale). Participants also indicated their marital status, highest level of education, yearly income, employment status, social class, religion, and UK region of residence and ethnicity. Relevant ethical guidelines were followed, and the research was approved through University of Oxford’s Central University Research Ethics Committee, with the reference number MS-IDREC-C1-2015-068 (R49911/RE003).

Participants were first informed about the aim of the study and provided with some background information about MMR and the MMR vaccination. The information provided was adapted from the NHS website on MMR vaccination. First, it was explained that:

[…] measles, mumps, and rubella […] can result in serious and sometimes fatal complications. Measles can lead to pneumonia, meningitis and other infections, and in the UK it causes death in at least 1 in 5000 cases. Mumps is a major cause of deafness, and catching rubella in early pregnancy causes death or serious disabilities in unborn babies. UK children usually receive two doses of the MMR vaccine by the time they start primary school. However, some children miss one or both doses. One way to make sure that most children get the full course could be to offer MMR vaccine catch-up in secondary schools (age 11–16), alongside other vaccines that children routinely receive at this time. On the following page, we propose five different policies that describe how the MMR catch-up vaccine could be offered at secondary schools. Please read carefully through each policy and then on the next page indicate how much you support each of them.

Participants then read about each of the five vaccination policies. The policies were described exactly as above. Following these paragraphs, participants indicated their support for each policy on a scale from 1 “completely oppose” to 7 “completely support” (Policy Support). Participants also ranked each policy from 1 (best) to 5 (worst) (Policy Ranking). Next, they completed a range of items that measured their views about MMR, vaccinations, medicine, and health. Participants then provided demographic information.

## Results and Discussion

**Fig. 1:**
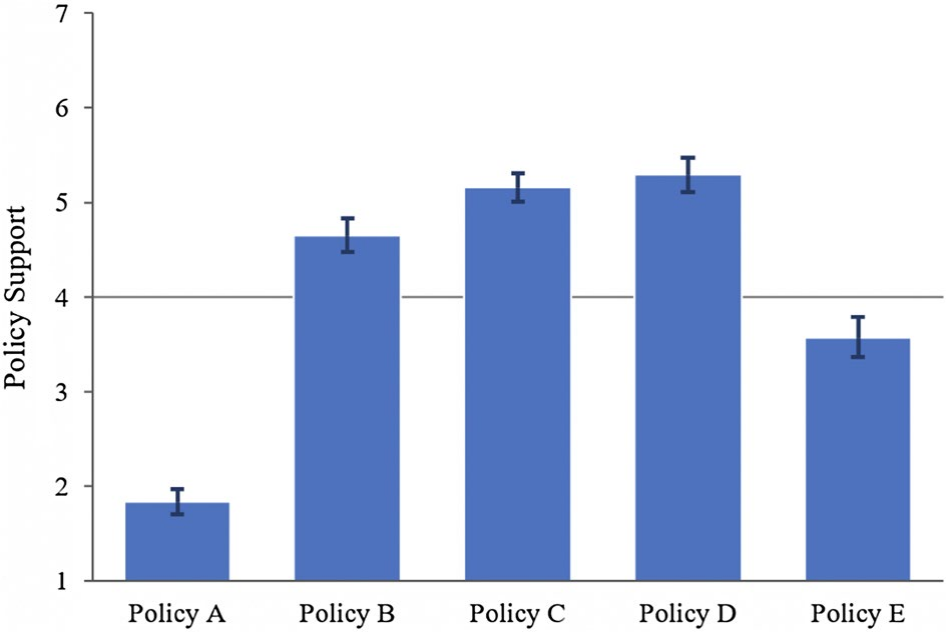
Policy A—no vaccination; Policy B—consent; Policy C—general permission; Policy D—opt out; Policy E—mandatory. Participants on average supported policies B, C, and D

Participants’ support differed significantly across policies: *F*(1, 456) = 274.39, *p* < .001, *ηG*^*2*^ = .33 (Fig. 1). On average, participants supported Consent (*M* = 4.65, *SD* = 1.89), General Permission (*M* = 5.16, *SD* = 1.60), and Opt-Out (*M* = 5.29, *SD* = 1.93), and opposed No Vaccination (*M* = 1.84, *SD* = 1.37) and Mandatory (*M* = 3.58, *SD* = 2.21). Support for General Permission and Opt-Out was not statistically different (*p* = .19), whereas support levels between all other policies were statistically different (for all other comparisons *p* < .001). In line with our prediction, support for Opt-Out was highest, followed by Consent and General Permission. Mandatory had markedly less support than Consent, with support for No Vaccination considerably lower again. There were no noteworthy correlations between policy support and demographic variables.

The results of this survey suggest that the overwhelming majority of UK citizens are likely to support some variation of offering of MMR vaccines in secondary schools. The two policies participants supported the most were making vaccination the default option in school (Default) and letting parents give general permission in advance (General Permission). These results suggests that UK citizens overall consider an opt-out policy to be an at least acceptable trade-off between high vaccination rates and respect for parent autonomy. Participants were almost as supportive towards a policy in which parents are asked to give explicit permission for the vaccination (Consent), which could be due in part to the fact that this is the current policy in the UK and therefore that parents of secondary school-age children in the UK are already familiar with this type of policy. Participants were largely against a policy that does not offer vaccines in secondary school at all (No Vaccination). However, they also on average slightly opposed mandatory vaccination in school (Mandatory). It is also noteworthy that while most participants were not in favour of mandatory vaccination in school, many were also not particularly opposed to it.

## Conclusions

In this paper, we have argued that one feasible, ethically acceptable, and potentially effective vaccination strategy is the use of vaccination nudges, and in particular making vaccination at school or day-care the default option and leaving parents the possibility to opt out if they so wish.

Default vaccination does not necessarily preclude other types of vaccination policies from being implemented in conjunction with it. In particular, it might be unfeasible, or perhaps unwise, to replace mandatory vaccination—where mandatory vaccination is already in place and has been proven to work—with a less restrictive policy such as the type of nudge we are proposing if this is likely to reduce total vaccination rates. If a restrictive and effective policy has come to be accepted by a large majority of a population, then the reason to replace that policy with a less restrictive one might be outweighed by reasons to avoid the risk of lower vaccination rates. In such contexts, there might even be a decisive case for implementing default vaccination *in addition to* mandatory vaccination policies in order to increase vaccination rates further. However, many countries, for example the UK, do not have mandatory vaccination policies but do experience problems of dealing with vaccine hesitancy and vaccine refusal, and therefore of keeping vaccination rates sufficiently high to confer herd immunity. This is particularly the case with MMR vaccine, given that measles requires a very high coverage rate in order for herd immunity to obtain. In these countries, if restrictive vaccination policies are to be introduced in order to increase vaccination rates or to ensure that vaccination rates remain high, it is preferable to start by introducing a type of policy such as vaccination nudges that are less restrictive than mandatory vaccination and consistent with the PLRA—again, assuming we accept that the PLRA represents a relevant constraint for vaccination policies. Whether or not nudges are effective in enabling a community to achieve herd immunity is an empirical question for which an empirical test is necessary. In this paper, we have suggested there are reasons to think that vaccination at school as the default option would not only be ethical but also effective because of decision biases that already affect many vaccination decisions.

## Footnotes

1 Though this is not necessarily the case: if a policy forces an individual to do what they would autonomously do anyway, the limitation of freedom, which depends on external constraints, does not entail a limitation of autonomy in the sense we have defined it here, at least if the reason why the individual chooses to abide by the policy requirement is their initial desire and not the desire to obey the law.
